# Controlled SrR Delivery by the Incorporation of Mg Particles on Biodegradable PLA-Based Composites

**DOI:** 10.3390/polym13071061

**Published:** 2021-03-28

**Authors:** Ana Ferrández-Montero, Alvaro Eguiluz, Elena Vazquez, Joab David Guerrero, Zoilo Gonzalez, Antonio Javier Sanchez-Herencia, Begoña Ferrari

**Affiliations:** 1 Institute of Ceramic and Glass (ICV), Spanish National Research Council (CSIC), 28049 Madrid, Spain; alvaro.eguiluz@icv.csic.es (A.E.); elenaa_v97@hotmail.com (E.V.); 4binfoguerrero@gmail.com (J.D.G.); zgonzalez@icv.csic.es (Z.G.); ajsanchez@icv.csic.es (A.J.S.-H.); bferrari@icv.csic.es (B.F.); 2 Laboratory of Physicochemistry of Polymers and Interfaces (LPPI), CY Cergy Paris University, Neuville-sur-Oise, 95031 Cergy, France; 3 Inorganic Chemistry and Chemical Engineering Department, University of Córdoba, Campus de Rabanales 14071 Córdoba, Spain

**Keywords:** drug delivery, strontium ranelate, magnesium, PLA/Mg, bone regeneration

## Abstract

Among several ions playing a vital role in the body, Sr^2+^ and Mg^2+^ are involved in the mechanism of bone formation, making them especially useful for bone tissue engineering applications. Recently, polylactic acid (PLA)/Mg composites have emerged as a promising family of biomaterials due to their inherent biocompatibility and biodegradability properties. In these composites, polymer and bio-metal have a synergetic effect—while the PLA inhibits the Mg fast reactivity, Mg provides bioactivity to the inert polymer buffering the medium pH during degradation. Meanwhile, the typical form of administrating Sr^2+^ to patients is through the medication strontium ranelate (SrR), which increases the bone mineral density. Following this interesting research line, a new group of composites, which integrates Mg particles and SrR charged onto halloysite nanotubes (HNT) in a polymeric matrix, was proposed. PLA/Mg/SrR–HNT composites have been processed following a colloidal route, obtaining homogenous composites granulated and film-shaped. The drug delivery profile was evaluated in terms of in vitro lixiviation/dissolution paying special attention to the synergism of both ions release. The combination of two of the most reported ions involved in bone regeneration in the composite biomaterial may generate extra interest in bone healing applications.

## 1. Introduction

Nowadays, one of the most studied strategies to improve and design new biomaterials is based on ion release, which controls and enhanced the biological response of the tissue. In the case of bone healing, several authors have described the critical effect of several essential elements or ions such as B^+3^, Mn^2+^, Mg^2+^, Sr^2+^, Ca^2+^, Zn^2+^ [1]. Strontium can be especially highlighted because similar to calcium, it regulates the osteoblast function when incorporated into the mineral phase of the bone [2]. A form of administrating Sr^2+^ is through the ranelic acid salt, strontium ranelate (SrR). This compound significantly improves the bone mass and its quality, increasing bone strength through changes in the matrix properties and bone mineral density in patients [3,4]. Several studies have informed about the procedures to load bio-composites with SrR, studying the drug delivery behavior and its associated biological response. The SrR dosage has been studied in polymer composites, in which SrR was directly dispersed in a biodegradable matrix. For instance, Deepthi et al. [5] reported a study about the SrR loading in a chitosan/alginate/fibrin gel as a potential candidate for cartilage regeneration. This composite only released 35% of SrR after 10 days, while 65% amount of SrR remained in the composite for a longer time. In the case of the biopolymer matrix, Loca et al. [6] described an in vitro study of the SrR/polylactic acid composite, indicating that the first 24 h, 15% of SrR was released. After that, 80% of drug release was completed within 121 days. Zhang et al. [7] also reported the fabrication of 3D printed polycaprolactone (PCL)/hydrogel composite scaffolds loaded with SrR. The in vitro release studies demonstrated that SrR has a significant fast release, more than 70% of the total drug being released after one day, after which an SrR-sustained release maintains for at least 21 days.

Another strategy to incorporate the SrR in biomaterials is by adsorbing it onto carriers or nanocarriers surfaces, as laponite or halloysite nanotubes. Nair et al. [8] reported about the preparation of polycaprolactone–laponite composite scaffolds for the controlled release of SrR, achieving cumulative release percentages of around 40% of the total drug load after 21 days. Abdollahi Boraei et al. [9,10] demonstrated that SrR delivered from halloysite nanotubes as cargo improves the proliferation of the MS cells and accelerates the osteogenic differentiation, hence enhancing bone formation and vascularization. In these cases, between 66% and 80% cumulative release of SrR was achieved after 21-days soaking in phosphate buffer solution (PBS).

In addition to Sr^+2^, magnesium and Mg^2+^ ions have special interest since they are essential in some metabolic processes, such as protein synthesis or muscle function, and play a vital role in bone formation [11]. Furthermore, the Mg^2+^ ions also present an important role in bone maturity and elasticity, and their release has a significant effect on osteogenesis. Several authors have described the effect of Mg^2+^ release on accelerating bone healing due to the stimulation of local cells around biodegradable magnesium implants [12]. According to this, recently it has been demonstrated the feasibility of including Mg particles within a polymer matrix such as polylactic acid (PLA) [13,14,15]. It has been proved that Mg promotes in vitro cell proliferation and the initial induction of osteogenesis [16,17,18,19,20] and reduces biofilm formation [19]. In addition, Mg improves composite mechanical properties [18,21], accelerates the degradation rate [22,23], and prevents the acidification of the medium resulting from the degradation of the polymer [24]. Additionally, it has been analyzed how Mg^2+^ ions interact with strontium ranelate increasing ALP activity, the enzyme responsible for bone formation and mineralization. The synergistic effect of Mg^2+^ and the drug is defined as an alternative method to regulate bone matrix growth and mineralization [25]. In fact, comparative studies have reported the different behavior in terms of cell culture that Sr^2+^ and Mg^2+^ ions produce in the bone; therefore, both ions should be considered as complements of each other and not competitors. For example, the inclusion of Mg^2+^ in coatings produces an improvement of cell attachment and proliferation, while the presence of Sr^2+^ induces the acceleration of bio-mineralization [26]. However, in terms of drug delivery, there is still a lack of information about how an external agent as Mg, a degradation accelerator, could affect the Sr^2+^ ions release in a biomaterial.

Among the most cutting-edge preparation strategies for composite biomaterials, the colloidal route is an excellent alternative since it allows controlling the dispersion of the particles in the polymeric matrix during shaping, which enhances the final properties of the microstructures [27,28]. The colloidal approach is based on the control of the interparticle forces in the liquid media, which implies significant advantages in terms of correct phase distribution and final homogeneity of a composite. The manipulation of the colloidal chemistry in the suspensions of inorganic particles in polymers solutions is the keystone to break down weak agglomerates, stabilize, and facilitate particle mixing, removing defects that degrades the relevant properties of the biomaterials [29]. Moreover, the use of processing additives helps to tailor other important parameters, such as the viscosity, flow, surface tension, wettability, etc., adapting the flux of the suspension to the manufacturing technique while controlling the arrangement of the particles in a polymeric matrix. Suspensions’ tailoring has been demonstrated to provide versatility in shapes and compositions during biomaterial processing.

In this study, a colloidal procedure is proposed to prepare composite materials in which halloysite nanotubes are used as cargo for SrR and both species, the SrR–HNT and the Mg particles homogeneously mixed and dispersed in suspension to produce PLA-based bio-composites. Both the amount of incorporated Mg and its degradation capability in aqueous media were considered in order to control the drug release rates. Composites were manufactured in two different shapes—granules and films = by drying under vacuum and using a tape casting method, respectively. Finally, the release rates of both essential cations (Sr^2+^ and Mg^2+^) are studied using in vitro assays.

## 2. Materials and Methods

### 2.1. Preparation of Colloidal Suspensions

Materials included Mg particles (Mg > 99.8%, d50 = 29 µm, Nitroparis, Castellón, Spain), polyethylenimine (PEI pure < 1% water, pKa 8.6, Mw 25,000 mol/g, Sigma-Aldrich, Darmstadt, Germany), tetramethylammonium hydroxide (TMAH, 25 wt.% in water, Sigma-Aldrich, Darmstadt, Germany ) halloysite nanotubes (HNT, Sigma-Aldrich, Darmstadt, Germany ), strontium ranelate (SrR, PROTELOS, Servier Company, Suresnes, France), tetrahydrofuran (THF, 99% of purity Panreac, Darmstadt, Germany), and polylactic acid solution (PLA polymer 2003D with D-isomer content of 4.25% provided by Nature Works, Minnetonka, Minnetonka, MN, USA). Instruments included scanning electron microscope (SEM, Hitachi TM-1000) (Hitachi Co, Tokyo, Japan), UV–Vis spectroscopy (Perkin Elmer UV–vis Spectrometer Lambda 950) (PerkinElmer, Waltham, MA, USA), inductively coupled plasma–optical emission spectroscopy (ICP–OES, Thermo Jarrell Ash, Franklin, MA, USA), direct reading ICP emission spectrometer (Thermo Jarrell Ash, Franklin, MA, USA), and Fourier transform infrared spectroscopy equipped with a total attenuated reflectance device (ATR–FTIR, Perkin Elmer) (PerkinElmer, Waltham, MA, USA).

Individual colloidal suspensions of magnesium (Mg) and halloysite nanotubes–strontium ranelate (HNT–SrR) were prepared in order to modify their particle surfaces achieving very high homogeneity in the formulation of compound materials. On one hand, as-received Mg particles were stabilized at pH 11–12 in deionized water at 35 vol.% by adding 0.2 wt.% (referred to powder) of a cationic dispersant polyethylenimine (PEI). Tetramethylammonium hydroxide was used to adjust the pH values in the basic range. Modified Mg particles by PEI coverage were then ball-milled for 45 min (using Nylon balls). The milling time was adapted to homogeneously disperse Mg particles in the suspension, without effect on its morphology and its particle size. In this sense, the agglomeration of particles was additionally avoided maintaining final sizes around 29 µm. The complete procedure used to optimize the particle dispersion and adsorption of PEI onto Mg particle surfaces has been previously described elsewhere [13]. On the other hand, HNT was used as cargo of the SrR. A total of 10 g/L HNT were dispersed in distilled water at pH 7. HNT was charged with 3 wt.% of SrR and additionally stabilized by adding 2 wt.% of PEI and stirred overnight. Detailed information about the cargo morphology, charge procedure, and its subsequent stabilization was previously supplied elsewhere [9,10].

**Figure 1:**
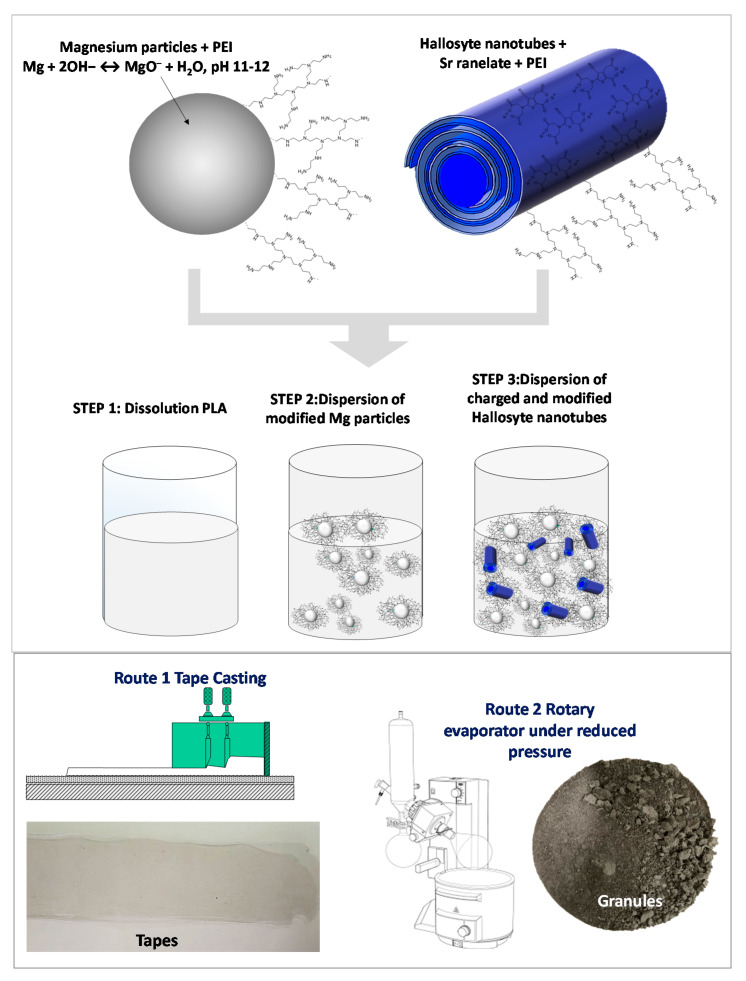
Scheme of the fabrication procedure of films and granules.

PLA solution was prepared by dissolving pellets of the polymer in tetrahydrofuran under magnetic stirring. Two different Mg contents (5 and 10 wt.%) were chosen to formulate the composites. Both compositions were prepared by adding the necessary amount of modified Mg–PEI in a certain volume of a PLA solution. The corresponding amount of modified HNT–SrR–PEI was then added to the PLA or the Mg/PLA composites dissolved in THF. All components were mixed under vigorous mechanical stirring. Formulated composites are labeled and summarized in Table 1. Figure 1 provides visual details of the followed steps during the preparation procedure of the composites.

### 2.2. Granules and Films Preparation

The Mg–HNT–SrR suspensions were then processed in order to obtain both, tape-cast films and granules of dried powder (Figure 1). Suspensions contained 5 or 10 wt.% of Mg (modified with 0.2 wt.% of PEI referred to the Mg mass) and 4 wt.% of HNT (with a 3 wt.% of SrR and 2 wt.% of PEI, both referred to the mass of HNT). Suspensions were dried under reduced pressure, and resulting granules were preserved at 60 °C. Suspensions were also cast to obtain the films. The solvent casting technique was performed on a glass plate using a tape casting device with a moving container. The thickness of the deposited film was controlled by the gap between glass, and two blades were attached to the container with micrometric screws. The casting parameters were 10 mm‧s^−1^ of carrier speed and 100 μm of gap height between the blades and the carrier tape. The resulting films were also preserved at 60 °C to avoid changes in the polymeric matrix.

### 2.3. Granules and Films Characterization

The microstructure of the granules and as-prepared films were characterized before and after drug releasing with a tabletop scanning electron microscope.

In vitro degradation behavior was studied in triplicate tests by immersion of 0.5 g of granules or films in phosphate buffered saline solution (PBS) in a proportion of 20 g/L at 37 ± 1 °C. All samples were shaken in an orbital shaker at 40 rpm. Although PBS does not accurately simulate body conditions, it is suitable for comparative studies on the hydrolytic degradation of PLA-based materials. The amount of SrR released and the pH evolution were recorded at different times. The SrR was quantified using UV–Vis spectroscopy. Total Sr^2+^ concentration at each time point was calculated from absorbance values of the released solutions considering a volume of media (mL) to specimen surface (cm^2^) ratio of 20:1. The solution was renewed regularly with a fresh medium after each sampling. Each time point is represented as the average of the triplicate test per specimen with the average standard deviation. Details of the experimental setup can be found elsewhere [13].

After soaking granules and films, a PBS aliquot was taken to measure Mg^2+^ ion concentrations released by using inductively coupled plasma–optical emission spectroscopy.

After release analyses, the evolution of the compound powders and films were evaluated by Fourier transform infrared spectroscopy (ATR–FTIR). An FTIR spectrometer equipped with a total attenuated reflectance device (ATR) was used to obtain the IR spectra of the samples in the region of 4000 to 500 cm^−1^. The results were shown in transmittance (%) and normalized to the highest peak intensity for their comparison.

## 3. Results and discussion

### 3.1. Drug Delivery in Composite Granules 

An intensive characterization of the particles herein used has been previously reported elsewhere [9,10,13,19]. HNTs are aluminosilicate sheets rolled up similar to a scroll with an external siloxane surface and an internal aluminol surface. The HNT surface exhibits a negative charge for all pH ranges since it can be roughly described by a superposition of mostly negative surface potential of the SiO_2_ exposed at faces and edges of the halloysite sheets with a small contribution from the positive inner surface of the Al_2_O_3_. Abdollahi Boraei et al. [10] described that the addition of 3 wt.% SrR to the HNTs suspensions at pH 7 neutralizes the strong negative charge of HNT surfaces (−65 mV) by the specific adsorption of Sr^2+^ ions up to achieve zeta potential values of −30 mV, while the subsequence addition of 2 wt.% PEI stabilizes the cargo–drug system in aqueous media reversing the surface charge sign up to reach + 40 mV. Changes in the surface charge confirmed that both molecules, the SrR and PEI, are at least electrostatically linked to the (HNT) cargo. Following the protocol described by Ferrández-Montero et al. [14], stabilized HNT–SrR was suspended in a PLA solution and, as it was described above, PLA/HNT–SrR composites were obtained. In a similar way, Mg-based composites were prepared by mixing the HNT–SrR species and 5 or 10 wt.% of the PEI-modified Mg particles [13] to prepare the PLA/Mg/HNT–SrR composites. The composition of 5 or 10 wt.% of Mg particles was selected since both were reported in the literature as the best equilibrium between a controlled degradation and better cell viability and proliferation in vitro results [19]. In all cases, the content of the HNT–SrR was set at 4 wt.% of the composite formulation of the granule, varying the PLA/Mg ratio since the objective is to evaluate the effect of the degradation agent in the release rate of the drug. A 4 wt.% HNT–SrR content was selected since it has been proved that the release profile of this drug concentration promotes stem cell proliferation, osteogenic differentiation, and enhancing bone regeneration [10]. Both species, HNT–SrR nanoentities and Mg particles, were stabilized by PEI adsorption, which also increases the compatibility with the PLA matrix by direct interaction. The surface charge of Mg particles varied from −40 mV to 15mV after PEI adsorption favoring the electrostatic link between the amine groups of the modified Mg particles and the hydroxide groups of the PLA matrix during the composite granulation. This phenomenon has already been defined in previous studies [14]. 

**Figure 2:**
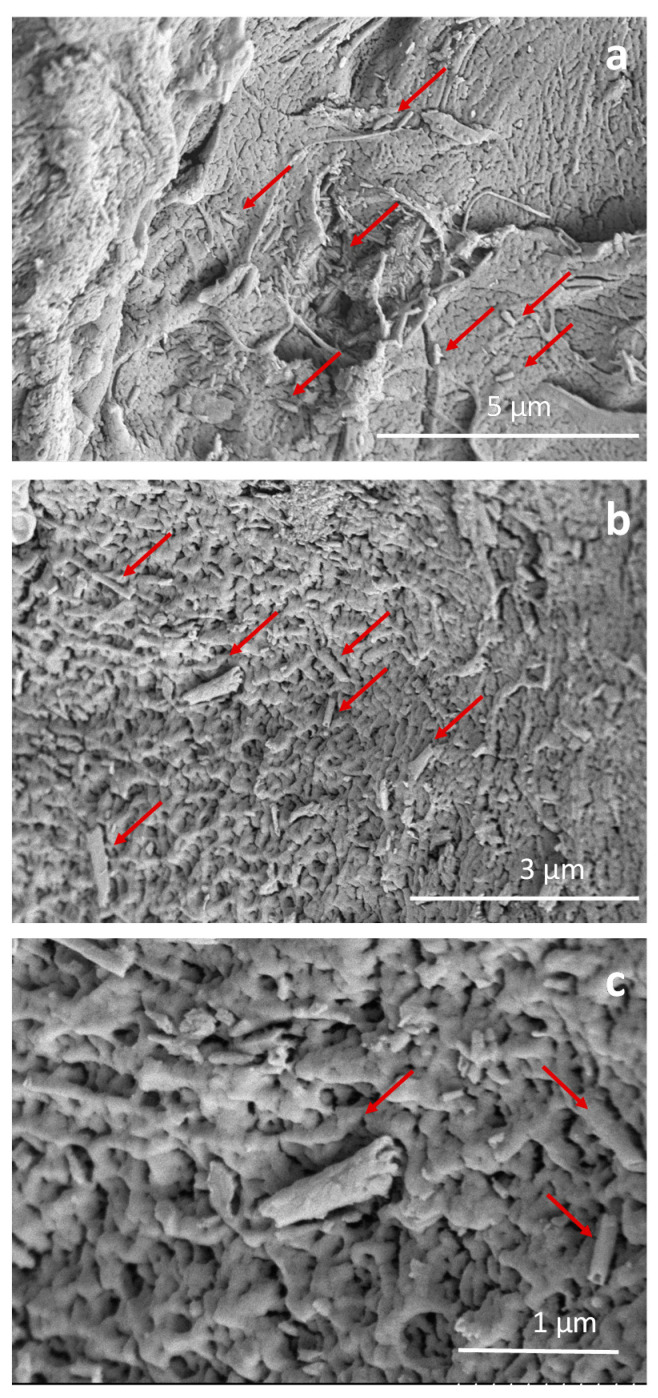
Micrographs of G-HNT granules at different magnifications, highlighting the dispersion of halloysite nanotubes–strontium ranelate (HNT–SrR) (red arrows) (**a**,**b**) and the nanotube morphology (**c**).

**Figure 3:**
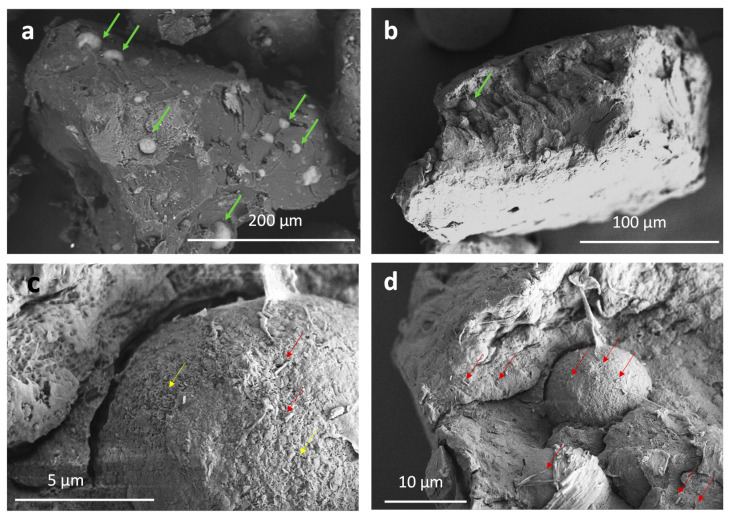
Micrographs of G-10Mg granules at different magnifications, highlighting (**a**,**b**) Mg particles (green arrows), (**c**,**d**) HNT–SrR (red arrows) and (**c**) the formation of MgO/Mg(OH)_2_ nanoplatelets (yellow arrows).

Formulated composites shaped in granules fit the bone filling features. SEM inspection of G-HNT and G-10Mg granules are shown in Figure 2 and Figure 3, respectively, to evidence the dispersion of HNT–SrR achieved in the prepared composites.

Micrographs in Figure 2 show HNT–SrR (red arrows) imbibed in the PLA matrix inside of G-HNT granules. In Figure 2a, it can be observed that cargo–drug nanoentities are very well dispersed along the PLA matrix. Figure 2b shows a detail of its dispersion. Most of the HNT–SrR particles are isolated and uniformly dispersion in the biodegradable matrix. Finally, the micrograph in Figure 2c shows in detail the nanotube features.

In Figure 3, the microstructure and morphology of G-10Mg granules are shown. Micrographs in Figure 3a,b shows the Mg particles (green arrows) of 20–10 µm in diameter, imbibed into the PLA matrix of the granules. Additional EDX analysis reported previously elsewhere (here not included) confirmed the presence of Mg. Nevertheless, a close inspection reveals the surface of the Mg particles and the inner-microstructure of the composite (Figure 3c). The red arrows highlight the HNT–SrR laid on the surface of the Mg particles and inserted in the biopolymer. The detail of the Mg surface (Figure 3d) shows the formation of MgO/Mg(OH)_2_ nanoplatelets (yellow arrows), covering most of the Mg particles, as a collateral product of the composite processing [13]. 

**Figure 4:**
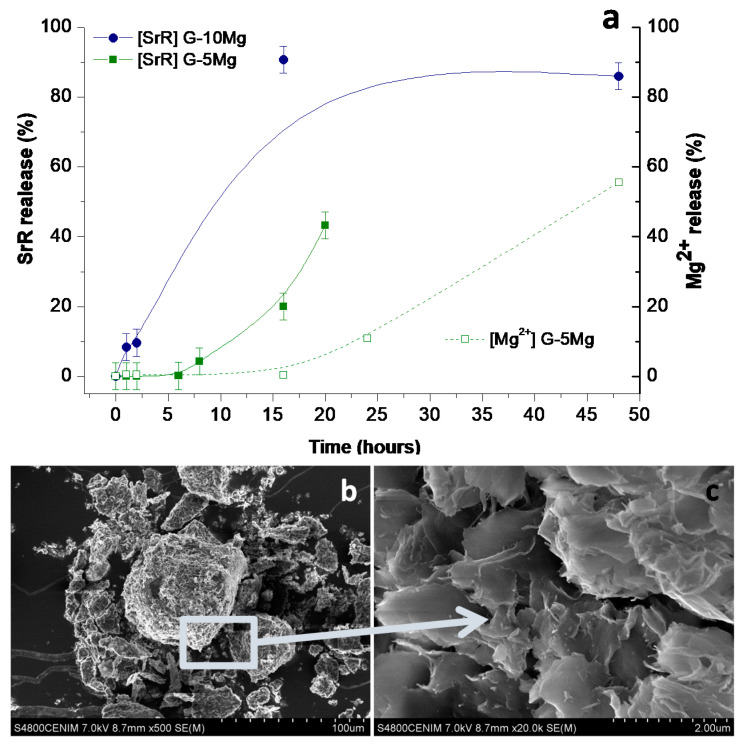
(**a**) Sr^2+^ and Mg^2+^ concentration determined by inductively coupled plasma (ICP) in the immersion medium during the degradation of G-5Mg and G-10Mg granules in phosphate buffer solution PBS and (**b**) Mg particle covered by polylactic acid (PLA) (**c**).

The plot in Figure 4a shows the data of the SrR and Mg^2+^ ions release during granules immersion in PBS up to 50 h. For G-5Mg granules, Sr^2+^ releases faster than Mg^2+^. Moreover, 43% of the SrR immobilized in the PLA matrix is delivered from the granules to the liquid medium after only 20 h of immersion, while double time is needed to achieve a similar Mg^2+^ ions concentration in the PBS. The lability of the SrR inside the PLA matrix, even when it is linked to the (HNT) cargo, leads to a faster release in granules than the Mg^2+^ ions liberation coming from imbibed Mg particles dissolution. Mg micro-particles are not so accessible to PBS since they are fully covered by a layer of PLA, and their specific surface is lower and less prone to react than adsorbed SrR on the nanotubes’ surfaces. The layer of PLA surrounding a large and isolated Mg particle observed in Figure 4b offers evidence of how after granulation of the colloidal mixture, PLA is fully covering each Mg particle.

Differences in the release behavior of Mg^2+^ and SrR show that the Sr^2+^ liberation is not related to the Mg particles’ dissolution for the granules G-5Mg composites. Although the amount of drug is similar in both composites, in the G-10Mg granules, the Sr^2+^ liberation is faster than from G-5Mg composites. This behavior could be related to the faster degradation of the granules with a double amount of Mg particles [19] since, as the amount of Mg increases from 5 to 10 wt.%, the particles’ dispersion doubles the reacting surfaces, and the release steps up.

**Figure 5:**
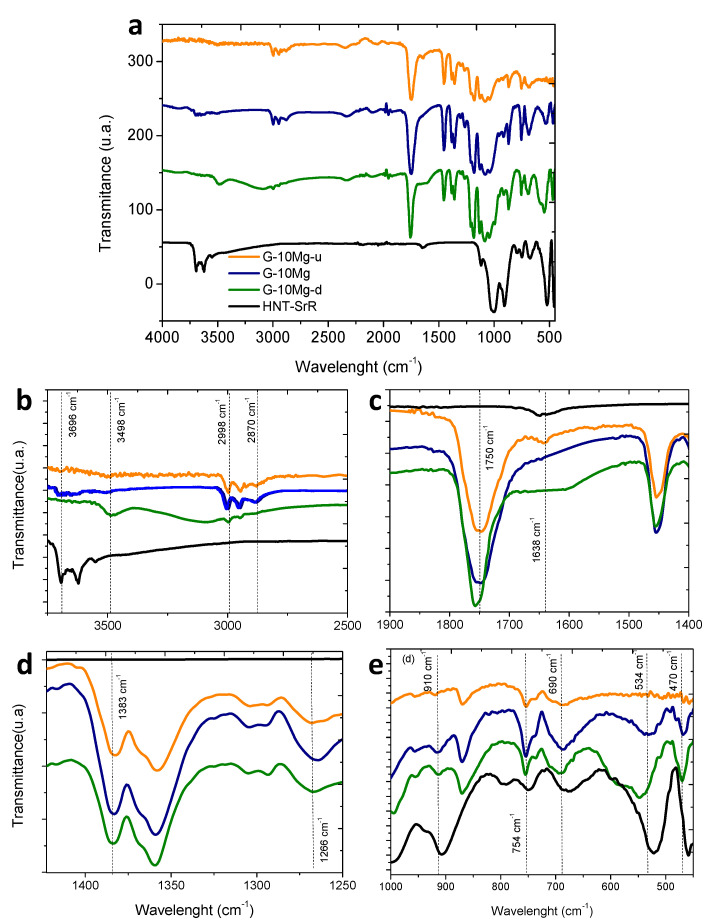
Depicts the complete FTIR spectra of HNT-SrR, G-10Mg-u, G-10Mg, and G-10Mg-d granules (**a**) and some representative bands associated (**b**,**c**,**d** and **e**).

After the study of drug release in PBS, the physicochemical analysis by FTIR–ATR was carried out in order to determine potential relationships among the release of the different species at the composite, and the changes produced by the addition of the HNT–SR to the PLA/Mg granules. Additionally, the chemical degradation produced by composite immersion in PBS was determined. The G-10Mg granules were considered for this study in order to maximize the signal to better follow the evolution of the composite. Figure 5a depicts the complete FTIR spectra of the granules with 10 wt.% of Mg particles, drug charged (G-10Mg) and uncharged (G-10Mg-u), and the drug-charged and degraded composite (G-10Mg-d) for 50 h. Representative bands associated with the different compounds are shown in Figure 5b–e for discussion of the results.

The composite presents the main bands of the raw materials, the matrix (PLA), the inorganic particles (Mg and HNT), the drug (SrR), and the stabilizer (PEI). Some bands are overlapped. In the FTIR detail for large wavelengths in Figure 5b, a peak of PLA attributed to the carboxylic acid group (3498 cm^−1^) only appears in the degraded sample, G-10Mg-d, and can be associated with the degradation of the polymer chain during the immersion of the composite. The peaks at 2998 and 2946 cm^−1^, related to CH stretching of the main PLA chain, also decrease with degradation. Moreover, the peak at 2850 cm^−1^, attributed to the CH_2_ stretching, is first displaced at 2870 cm^−1^ for HNT–SrR charged G-10Mg composites, and then it disappears after the degradation of the materials. The most characteristic band of PLA at 1750 cm^−1^ (Figure 5c), which is attributed to the carboxyl bond of the ester group, is extremely sensitive to changes caused by polymer degradation, either by decreasing its intensity or by shifting its wavelength downwards [30], as occurs in the degraded composite. In Figure 5d, the band at 1383 cm^−1^ attributed to the CH_3_ group is slightly displaced in the degraded composite (G-10Mg-d). Additionally, the peak at 1266 cm^−1^ linked to the terminal -OH bonds of the ester group, increases with the drug presence (G-10Mg), to later decrease its intensity in the degraded material (G-10Mg-d). This can be related to a low interaction between the drug and the polymer, which can decrease after the composite immersion.

Related to the Mg bands, in Figure 5b, the bands above 3696 cm^−1^ are observed in the composite G-10Mg and G-10Mg-u, and they are associated with the hydroxyl bond of the Mg-OH group [31]. The presence of this peak is related to the MgO/Mg(OH)_2_ layer, which is covering the surface of the Mg particles (see Figure 3c). 

Additionally, in the detail of the FTIR spectrum in Figure 5e, bands related to HNT, which overlapped with PLA ones (754 and 690 cm^−1^), are shown. The HNTs exhibit a bending vibration of Al–OH at 910 cm^−1^, the Si–O perpendicular stretching at 754 cm^−1^ and 690 cm^−1^. The band observed at 534 cm^−1^ corresponds to the vibration of Al–O–Si, and the band at 470 cm^−1^ corresponds to the deformation of Si–O–Si- [32]. The most highlighted result is the unusual displacement of the 534 cm^−1^ bands after the degradation, which is related to a change in the HNTs provoked by the SrR release. Finally, the characteristic peaks of SrR at 1083 and 1317 cm^−1^ bands overlap with PLA bands, and could not be associated in the FTIR [5], but the shift at the PLA band 1638 cm^−1^, which was observed in G-10Mg-u and HNT–SrR, disappears in the G-10Mg and G-10Mg-d, revealing a weak interaction between the polymer and the HNT–SrR compound in the composites and probably also a residual amount of SrR in the degraded composite [6].

According to the results, the granules present a low degradation rate after the SrR release since the bands associated with PLA slightly displace, and its intensity decreases. Some changes observed in PLA bands of G-10Mg suggest the presence of a low interaction between the HNT–SrR and the polymer. Additionally, the displacement of some HNT bands, but not their disappearance, shows the presence of HNT in the degraded samples and the release of SrR.

### 3.2. Drug Delivery in Composites Films 

**Figure 6:**
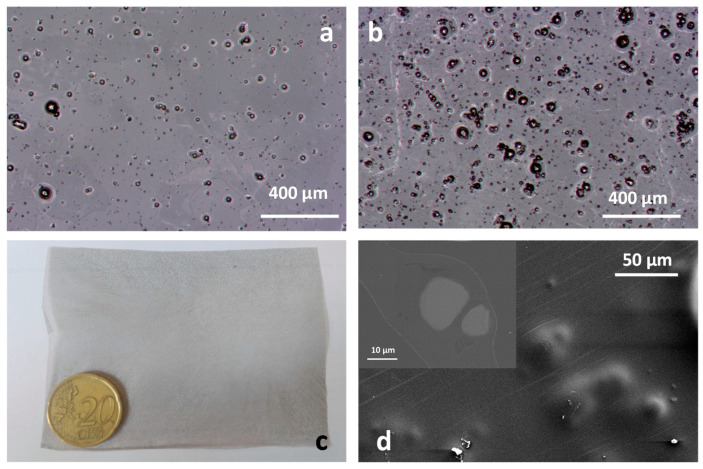
General view of the surface of F-5Mg (**a**) and F-10Mg (**b**) films, a macroscopic view of the film (**c**), and a detail of the surface of the imbibed Mg particles fully covered by the PLA (**d**).

The casting of formulated composite suspensions results in the film-shaped biodegradable membranes shows in Figure 6 (F-5Mg and F-10Mg films). A view of the F-5Mg and F-10Mg surfaces can be observed in Figure 6a,b, in which the dispersion of the Mg particles imbibed in the PLA matrix is shown. Particles ranging from 40 to 4 µm are randomly distributed in the film, offering evidence of the wide particle size distribution of the Mg powder used to fabricate those films. The picture in Figure 6c shows the general view of the processed composites. The high homogeneity and good particle dispersion produced by the colloidal approach are also herein confirmed. The SEM micrograph in Figure 6d shows that Mg particles are completely covered by the PLA and are not directly exposed to the PBS during the degradation tests. The detail in the inset shows the cross section of the F-10Mg film, corroborating the full coverage of the bioactive metal by the polymer in the biofilm. 

**Figure 7:**
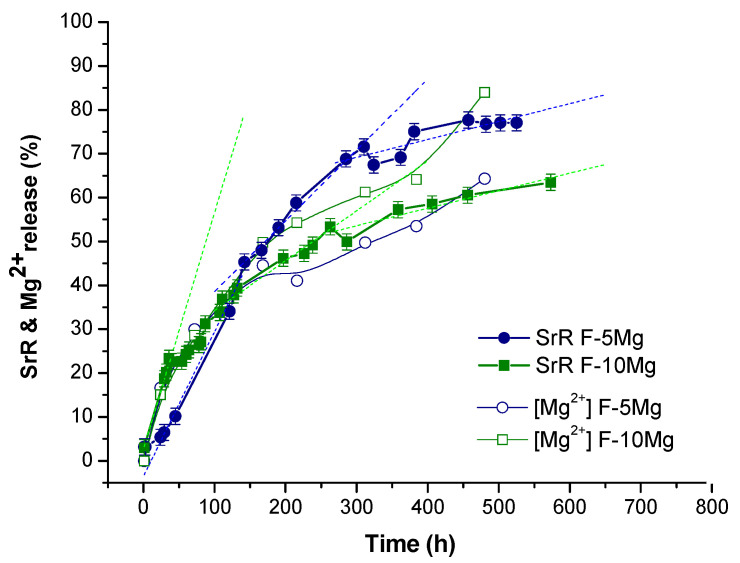
The cumulative release of SrR determined by UV–Vis spectroscopy and the Mg^2+^ concentration determined by ICP in the immersion medium during the degradation of F-5Mg and F-10Mg films in PBS.

In order to evaluate what differences emerge in the drug release profile due to the change of composite shape from granules to films, the drug delivery test was carried out for F-5Mg and F-10Mg. Figure 7 shows the evolution of Mg^2+^ lixiviation from the films and the SrR release determined by UV–Vis spectrometry during degradation in PBS. The Mg^2+^ liberation has been previously described in the literature for similar films, but in static conditions and in absence of the drug [19]. When Mg particles are dispersed and immobilized in the biodegradable polymer, an increase of the release of Mg^2+^ ions in relative terms during a first week (168 h) due to the film degradation is registered. The release decays after 28 days (336 h) since Mg particles practically dissolve in one month. In those terms, as expected, the higher is the Mg particles concentration in the film the faster is the Mg^2+^ release. 

In fact, in our composites (PLA/Mg/HNT–SrR), the Mg^2+^ liberation follows a similar trend being faster the liberation of Mg^2+^ for the films with a higher content of particles (F-10Mg). In F-5Mg films, 64% of Mg is dissolved and liberated to the liquid medium after 480 h. This amount increases in percentage, and hence in absolute value terms, reaching 84% during the degradation of the F-10Mg composite.

On the other hand, Abdollahi Boraei et al. [10] have recently described the relationship of the SrR release with the concentration of the distributed drug in a biodegradable matrix. The authors measured large differences in the relative SrR discharge (from 60% to 80% release after 336 h) as a function of the drug loading in the composite. In our case, both composites F-5Mg and F-10Mg were charged with a similar amount of drug, and consequently, differences in SrR release should be related to the Mg content in the composites.

In fact, the Mg^2+^ liberation follows a similar trend in accumulative values to SrR release in the PLA/Mg/SrR composites. Drug release during the first 120 h (5 days) is faster for F-10Mg than for F-5Mg films. This is related to the fast degradation of F-10Mg films during the first days of immersion. In fact, the F-10Mg film liberates 25% of the immobilized drug during the first 60 h, while the liberation of this amount of drug in F-5Mg films takes 100 h. However, this tendency changes when 40% of the drug was delivered and also 40% of Mg^2+^ was liberated. While 77% of the immobilized SrR is released in 575 h in the F-5Mg films, the F-10Mg composite liberates only 64% of its drug content due to the general behavior of the drug release apparently being independent of the Mg^2+^ liberation in the last stages of degradation. Table 2 shows a summary of extrapolated data in the SrR release curve for both films related to changes in the slope of SrR release kinetics. It is important to note that after 270 h and 310 h (11–13 days) drug delivery kinetics reduces for both composites, reaching similar values of SrR liberation rates (0.04% per hour). However, at this time, while the F-10Mg composite has delivered 52% of the immobilized drug, the F-5Mg composite has delivered 72%.

**Figure 8:**
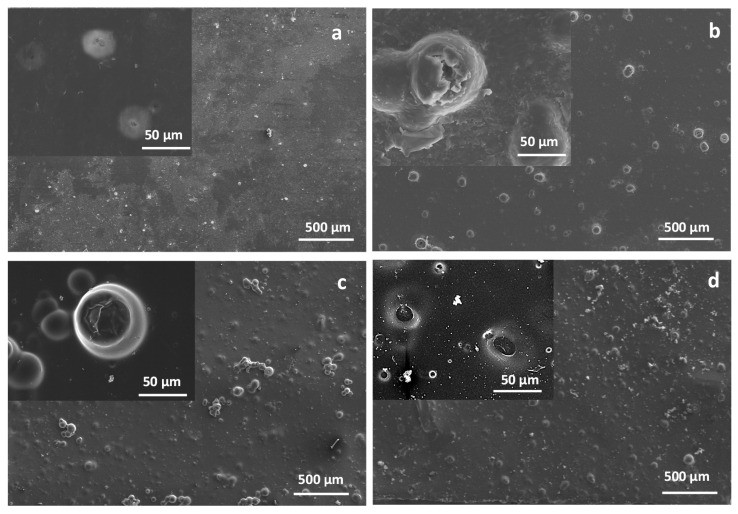
General view of the surface of F-5Mg films degraded for 100 h (**a**) and 265 h (**b**), and of F-10Mg films degraded for 170 h (**c**) and 500 h (**d**). At the insets, a detail of the degradation of Mg particles and surrounding PLA matrix is shown.

Micrographs in Figure 8 show the general views of the surface of F-5Mg films degraded for 100 h and 27% of SrR liberated (Figure 8a) and 265 h and 66% of SrR liberated (Figure 8b); of F-10Mg films degraded for 170 h and 43% of SrR liberated (Figure 8c) and 500 h and 62% of SrR liberated (Figure 8d); and details of the Mg particles degradation in each case.

The micrographs in Figure 8a show the smooth surface of the composite after the release of 27% of the drug immobilized in the F-5Mg film, where Mg particles are not visible in a general view of the film at lower magnifications. However, a detailed inspection of the Mg particles reveals the formation of small pits at the PLA surface due to the degradation activity of Mg over the PLA. For longer degradation times of this composite (265 h and the 66% of released drug), the Mg particles are clearly revealed at the film surface, and the degradation of the PLA surrounding the Mg particles step up. A similar aspect shows the degraded F-10Mg film after 170 h and 42% of the drug released, while after 500 h and 62% of the drug liberated, most of the Mg particles of the F-10Mg film are completely dissolved, as the holes in the inset of Figure 8d clearly indicate. 

The complex release mechanisms taking place in the PLA/Mg/HNT/SrR biocomposite impede the adjustment of the release profiles of both ions Sr^2+^ and Mg^2+^ to the mathematical models described in the literature [33,34].

SrR release reported in the literature shows that in a similar biopolymer, PCL, loaded with SrR, liberates 70% of the total drug after one day [7], while in a PLA matrix, only 15% of SrR is released during the first 24 h [6]. Using carriers or nanocarriers, such as the laponite or the halloysite nanotubes, the drug release can be controlled, delivering around 40% of the total drug after 21 days [8]. The PLA granules loaded with Mg and SrR–HNT present a similar release profile to other polymer matrices without carriers, delivering around 50–80% after one day and varying with the Mg content. However, the film-shaped biocomposite allows better control of the SrR release, delivering 60–70% after 21 days. These values are higher than those obtained for other composites with SrR loaded carriers, demonstrating that a reactive agent as Mg metal accelerates the global SrR release.

**Figure 9:**
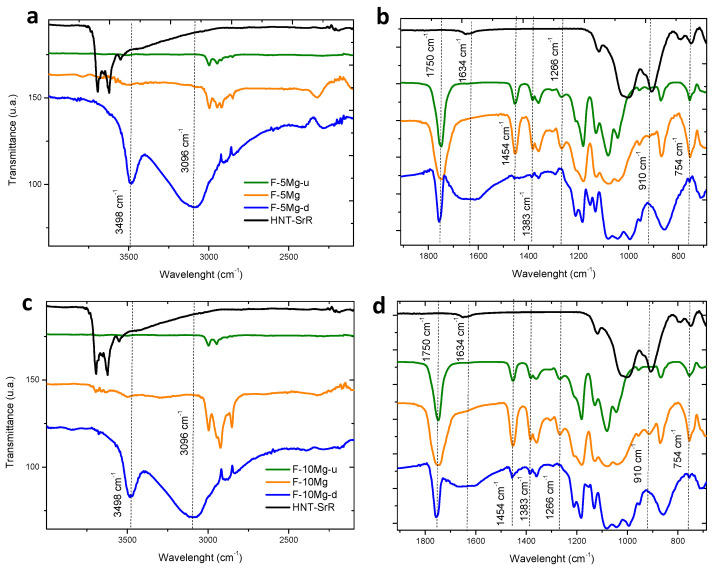
Depicts the complete FTIR spectra of F-5Mg-u, F-5Mg, and F- 5Mg-d (**a**,**b**) composites and F-10Mg-u, F-10Mg, and F-10Mg-d (**c**,**d**) films.

The FTIR measurement of the films was carried out in order to compare with the composite granules and analyze the degradation produced by immersion in PBS. Figure 9 depicts the FTIR spectra of films with 10 wt.% of Mg particles, drug charged (F-10Mg) and uncharged (F-10Mg-u), and the drug-charged and degraded composite (F-10Mg-d) for 575 h (Figure 9a,b), and similar samples for 5 wt.% of Mg particles—F-5Mg, F-5Mg-u, and F-5Mg-d (Figure 9c,d). The band at 3498 cm^−1^ in both composites, attributed to the carboxylic acid group, appears in the degraded films as a wide band with considerable intensity, compared with the composites before the immersion. This fact is associated with the large degradation of the polymer chain during the composite immersion, and in this case, significantly higher than in the granule composites. The same can be extrapolated to the peak at 3096 cm^−1^, which is related to the stretching of the OH group produced by PLA hydrolysis. The most characteristic band of PLA at 1750 cm^−1^ (Figure 9b,d), which, as was mentioned before, is extremely sensitive to changes caused by polymer degradation, presents a significantly less intensity for the degraded composite. The presence of SrR is evidenced by the small shoulder at 1634 cm^−1^ in F-10Mg (Figure 9d). The band widening at the G-10Mg-d spectrum corresponds to OH scissoring and suggests the PLA degradation. This fact is related to the ester bond reaction to form carboxylic acid groups.

Additionally, different bands of PLA, such as the band at 1383 cm^−1^ attributed to the CH_3_ group or the peak at 1266 cm^−1^ linked to the terminal -OH bonds, which are displaced or decreased their intensity in the granulated composite, disappear after the degraded films. Bands at 1454 cm^−1^ are related to PLA and also disappear during the degradation. Additionally, bands associated with the HNT, such as the bending vibration of Al–OH at 910 cm^−1^ and the peak at 754 cm^−1^, which correspond to the Si–O perpendicular stretching and could be also related to Mg interaction, also disappear after the immersion. These results show that the high degradation status of the composite induces the release of HNT and SrR. 

In conclusion, the inspection of the PLA structure by FTIR of F-5Mg-d and F-10Mg-d composites evidences their advanced degradation status. Moreover, FTIR analyses demonstrate that, although the PLA degradation is more evident in the films, the SrR delivery is faster in granules (1.2%/h for G-5Mg in Figure 4) as a consequence of the open structure of granules and the accessibility of the PBS to the internal structure and holes, which favors the species lixiviation. Species, Mg particles, and HNT–SrR are fully covered by PLA in the films, which retard their lixiviation during the film degradation. Consequently, the drug delivery can be better controlled in the films than in the granules. In fact, during the first stages of film degradation, drug delivery depends on the film degradation, catalyzed by the presence of the well-dispersed Mg particles. However, the features of the Mg particle degradation shown in the micrographs in Figure 8, and the elevated Mg^2+^ liberation highlighted in Figure 7 (evidence of the Mg particle dissolution) suggest the Mg^2+^ and Sr^2+^ have a competitive liberation. Consequently, although a priori, the Sr^2+^ liberation seems to be synergistic with the Mg^2+^ release; in above 40% of liberated Mg^2+^, the local concentration of Mg^2+^ ions is higher in the F-10Mg composite than in F-5Mg film. The higher local ion concentration provokes the reduction of the liberation of the labile Sr^2+^ ions and consequently reduces the kinetics of the drug delivery.

## 4. Conclusions

The simultaneous Sr^2+^ and Mg^2+^ ions release was evaluated by manufacturing a novel family of PLA-based composite biomaterials, processed in two formats shapes—granules and films. Both essential ions release profiles were evaluated to explore a potential synergistic release in PLA bio-composites. 

The SrR drug delivery can be better controlled in the films than in the granules. Results show that granules release 40–80% of the Sr^2+^ after only 20 h, depending on the Mg concentration in the composite. In the case of the films, both Sr^2+^ and Mg^2+^ degradation followed similar fast release during the first days. The 25% of Sr^2+^ was delivered between the first 60 and 100 h, depending on the Mg content. The stage of fast degradation is longer for the F-5Mg sample, and although the liberation tendency changes toward a slower kinetic for both Mg contents, F-5Mg films liberate 77% of SrR, whereas F-10Mg composites release only 64% after 21 days. Consequently, although the Sr^2+^ liberation seems to be related to the Mg^2+^ release during the early phase of film degradation, the increase of the local ion concentration leads to the competitive liberation of Sr^2+^ and Mg^2+^.

Differences found in the ions liberation in the two different shapes of the biocomposite, films, and granules, is as a consequence of the higher surface-exposed of granules, and the PBS accessibility, which favors the species lixiviation over the effect of the PLA degradation catalyzed by the Mg particles dissolution in the films.

Even though the SrR drug release values are in concordance with others reported in previous studies, the parallel liberation of the Mg and its effect on the drug delivery, and its role in bone healing may generate additional interest in the fabrication of these PLA/Mg/SrR composite types and their applications in bone filling and bone grafting.

## Figures and Tables

**Table 1:** Summary of the formulated composites.

Nomenclature	Composite Formulation and Feature (Weight Basis)
G-HNT	Granules of 96% PLA/4% HNT
G-5Mg	Granules of 91% PLA/5%Mg/4% HNT–SrR (3.88% HNT + 0.12% SrR)
G-10Mg	Granules of 86% PLA/10%Mg/4% HNT–SrR (3.88% HNT + 0.12% SrR)
F-5Mg	Films of 91% PLA/5%Mg/4% HNT–SrR (3.88% HNT + 0.12% SrR)
F-10Mg	Films of 86% PLA/10%Mg/4% HNT–SrR (3.88% HNT + 0.12% SrR)

**Table 2:** Parameters describing the SrR liberation kinetics for F-5Mg and F-10Mg composites.

F-10Mg			F-5Mg		
SrR Release (%)	Time (h)	Kinetics (%/h)	SrR Release (%)	Time (h)	Kinetics (%/h)
up to 23	38	0.53	up to 45	142	0.31
up to 52	270	0.11	up to 72	310	0.16
up to 64	575	0.04	up to 77	525	0.04

## Data Availability

The data presented in this study are available on request from the corresponding author.
